# Long-Term Effects of Placental Growth on Overweight and Body Composition

**DOI:** 10.1155/2012/324185

**Published:** 2012-04-18

**Authors:** Johan G. Eriksson, Jill Gelow, Kent L. Thornburg, Clive Osmond, Markku Laakso, Matti Uusitupa, Virpi Lindi, Eero Kajantie, David J. P. Barker

**Affiliations:** ^1^Department of General Practice and Primary Health Care, University of Helsinki, PL 20, 00014 Helsinki, Finland; ^2^Department of Chronic Disease Prevention, National Institute for Health and Welfare, PL 30, 00271 Helsinki, Finland; ^3^Vasa Central Hospital, Sandviksgatan 2-4, 65130 Vasa, Finland; ^4^Folkhälsan Research Centre, University of Helsinki, PB 63, 00014 Helsinki, Finland; ^5^Unit of General Practice, Helsinki University Central Hospital (HUS) 00029 Helsinki, Finland; ^6^Heart Research Center, Oregon Health and Science University, Portland, OR 97201-3098, USA; ^7^MRC Epidemiology Resource Centre, Southampton General Hospital, University of Southampton, Southampton SO16 6YD, UK; ^8^Department of Internal Medicine, Kuopio University Hospital, 70211 Kuopio, Finland; ^9^Department of Clinical Nutrition, Institute of Public Health and Clinical Nutrition, University of Eastern Finland, 70211 Kuopio, Finland; ^10^Department of Physiology, Institute of Biomedicine, University of Eastern Finland, 70211 Kuopio, Finland; ^11^Hospital for Children and Adolescents, Helsinki University Central Hospital, 00029 Helsinki, Finland; ^12^College of Science, King Saud University, Riyadh 11451, Saudi Arabia

## Abstract

Obesity is programmed in utero and small babies generally have small placentas. In some circumstances, an undernourished fetus can expand its placental surface to extract more nutrients. We hypothesize that this results in an imbalanced nutrient supply to the fetus leading to obesity. To determine whether placental size determines overweight and body composition, we studied 2003 subjects in adult life. Associations between placental surface area and indices of overweight were restricted to people who carried the Pro12Pro genotype of the *PPAR*γ*2* gene. For every 1 SD increase in placental surface area, the odds ratio for overweight was 1.37 (95% CI 1.10 to 1.71; *P* = 0.005). Expansion of the placental surface in compensation for fetal undernutrition increases the risk of overweight and a higher body fat percentage in people carrying the Pro12Pro genotype. We suggest that similar underlying multifactorial mechanisms affect the development of obesity in general.

## 1. Introduction

There is a body of evidence suggesting that type 2 diabetes is programmed in utero [1, 2]. Fetal programming is the process through which fetal malnutrition leads to lifelong changes in the body organs and systems in ways that might cause disease in later life [3]. There is some evidence that obesity, a major risk factor for type 2 diabetes, is also programmed in utero. Maternal hyperglycemia is associated with obesity in the next generation [4, 5]. Women who were in utero during the Dutch famine tended to be overweight as adults as a consequence of early programming [6]. Interestingly, animal experiments show that prenatal undernutrition upregulates appetite [7].

Fetal nutrition depends on the placenta ability to transport nutrients to the fetus from its mother [8]. This ability is reflected in its size [9]. Small babies generally have small placentas, but, in some circumstances, an undernourished fetus can expand its placental surface to extract more nutrients from the mother [10]. This phenomenon is well known to sheep farmers who induce placental expansion by undernourishing ewes in midgestation. When the ewes are returned to good pasture the expanded placenta results in a larger fatter lamb than they otherwise would be. There are findings suggesting that placental expansion occurs in humans through extension of the placental surface along its minor axis [11]. This is associated with long-term costs that include hypertension. Interestingly, in sheep placental expansion can only occur if the ewe was well nourished up to the time of mating [12].

Maternal body size, especially height, can be used as a marker of her life time nutrition. Short maternal stature is a product of poor fetal and childhood nutrition, or recurrent exposure to infections, and genetic factors [13]. The peroxisome-proliferator-activated receptor **γ*2* (*PPAR*γ*2) *gene encodes a nuclear hormone receptor that mediates adipocyte differentiation and regulates glucose and lipid metabolism [14–17]. Variants of the *PPAR*γ*2* have repeatedly been linked to overweight, insulin resistance, and type 2 diabetes. Furthermore, PPAR*γ* is known to play an important role in controlling placental vascular proliferation, trophoblast differentiation, and invasion [18, 19].

We have previously shown that the association between expansion of the placental surface with later hypertension is dependent upon maternal height [11]. We now speculate that the long-term costs could also include overweight and obesity in later life. We therefore examined the long-term effects of placental expansions on overweight and body composition taking maternal height and genetic factors, the *PPAR*γ*2 *gene, into account. We examined this in the Helsinki Birth Cohort Study (HBCS), which comprises people born in 1934–44 for whom the size of the placental surface was measured at birth.

## 2. Patients and Methods

The study cohort consists of 8760 men and women who were born between 1934 and 1944 in Helsinki University Central Hospital and who visited child welfare clinics in the city. Details of the birth records and child welfare clinic records have been described [20, 21]. The birth records included the mother's height. The weight and length of the baby at birth were recorded, and we calculated the ponderal index (birth weight/length^3^). The records also included the weight of the placenta, together with the maximal so-called “diameter” of the surface and a lesser “diameter” bisecting it at right angles. The diameters were measured because it was recognized that the placental surface is more oval than circular and the two diameters were used to describe this. Assuming an elliptical surface, we estimated the surface area of the placenta as maximal × lesser diameter ×*π*/4.

We used random number tables to select a sample of people within the cohort who were still living in Finland. In order to achieve a sample size in excess of 2000 people we selected 2902 subjects and invited them to a clinic, 2003 visited the clinic. The procedures used at the clinic have been described [21]. Written informed consent was obtained from each subject before any procedures were carried out. The Ethics Committee at the National Public Health Institute, Finland, approved the study. At the clinic height and weight were measured in light indoor clothing and without shoes on. Body mass index (BMI) was calculated as weight (kg) divided by height^2^ (m^2^). Estimates of total lean and fat mass were measured by bioelectrical impedance analysis using the InBody 3.0 eight-polar tactile electrode system, Biospace Co., Ltd, Seoul, Republic of Korea, as described [22]. Details of the genotyping procedure have been described previously [23].

### 2.1. Statistical Methods

We analysed overweight using multiple logistic regression and percent body fat and lean body mass using multiple linear regression. We always adjusted for age and gender in these regressions. The measurements of body and placental size were analysed as continuous variables. Tests for interaction used the product of the variables being studied.

## 3. Results

Table 1 shows the measurements of birth and placental size and current body size, together with the frequency of the Pro12Pro genotype.  Table 2 shows the odds ratios and regression coefficients for three outcomes, overweight (BMI ≥ 25 kg/m^2^), body fat percentage, and lean body mass, according to birth weight, ponderal index (birth weight/length^3^), and placental size. The odds ratios and regression coefficients represent the change in each outcome that is associated with a 1 SD increase in birth size or placental size. Lean body mass was predicted by each measurement of body and placental size. Overweight was predicted by high birth weight and high ponderal index, while percent body fat was predicted by high ponderal index. Neither overweight nor percent body fat was predicted by measurements of placental size.

As in previous analyses we divided the subjects around the mother's median height (160 cm) (Table 3). In both maternal height groups lean body mass was predicted by all measurements of body and placental size. Among people whose mothers were tall a long lesser placental diameter predicted both overweight and percent body fat. There was a statistically significant interaction between the effects of mother's height and the lesser diameter on percent body fat (*P* for interaction = 0.02). Among people whose mothers were short, no measurements of placental size predicted overweight or percent body fat.  Table 4 is therefore confined to people whose mothers' were tall. The subjects are divided according to their *PPAR*γ*2* genotype. Among carriers of the Ala allele overweight was predicted by a large maximal diameter but there were no other associations between placental size and either overweight or percent body fat. Among people with the Pro12Pro genotype large placental area and a long lesser diameter predicted both overweight and percent body fat (Table 4). There were statistically significant interactions between the genotypes and the effects of placental area and the lesser diameter on overweight and percent body fat (*P* for interaction = 0.004 and 0.05 for area and 0.03 and 0.09 for the lesser diameter, resp.). In people with the Pro12Pro genotype a long maximal diameter predicted overweight but not percent body fat. In a simultaneous regression with the lesser diameter the maximal diameter no longer predicted overweight. The results in Table 4 were similar in men and women.

## 4. Discussion

In the whole study sample placental size was not associated with either overweight or a high percent of body fat. We found, however, that an expanded placental surface and a long lesser diameter predicted overweight and high percent of body fat in a subset of men and women whose mothers were tall and who carried the Pro12Pro genotype of the PPAR*γ*2 gene. Higher birth weight was associated with an increased risk of having a BMI greater than 25 kg/m^2^ and with a greater lean body mass. This has been shown before and suggests that birth weight influences adult body mass index through its effect on lean body mass [22, 24]. Our findings suggest that lean body mass is related to the volume of placental tissue, reflected in its weight, while fat mass is related to placental surface area.

We have previously shown that an enlarged placental surface is associated with later hypertension, but this association was confined to people whose mothers were tall [11]. We interpreted this as evidence that compensatory placental expansion in humans is similar to compensatory expansion in sheep, in that it can only occur in women who were well nourished before they conceived. We have shown that people who had an enlarged placental surface and later hypertension had above-average birthweight [11]. This is consistent with sheep farming practices in which placental expansion is induced by undernourishing ewes [10, 12]. This leads to larger fatter lambs than would otherwise be. The association between a large placental surface and later hypertension depended on a large lesser diameter rather than a large maximal diameter [11]. Our findings for overweight and percentage body fat are similar in that they are only predicted by large lesser diameter. This is a further evidence that tissue along the minor axis of the placental surface is qualitatively different to tissue along the major axis [25]. Tissue along the minor axis may be more nutritionally sensitive.

We suggest that placental expansion increases the nutrient supply to the fetus, but this supply is unbalanced. We have previously proposed that compensatory placental expansion increases glucose transfer to the fetus, but this may not be matched by transfer of other nutrients, including proteins [26, 27]. Glucose crosses the placenta by diffusion whereas protein is actively transported. Placental enlargement could affect the fetus in the same way as high circulating maternal glucose concentrations, initiating biochemical changes that ultimately lead to obesity [4]. Our findings suggest that this only occurs in people who are homozygotes for the Pro12 allele of the PPAR*γ*2 gene. This allele is known to be linked with insulin resistance [14–17].

### 4.1. Limitations of the Study

We have previously discussed limitations of the Helsinki Birth Cohort Study [20, 21]. The data are restricted to subjects who were born in Helsinki University Central Hospital and attended voluntary child welfare clinics, did not emigrate, and were still alive and willing to participate in the year 2003. However, we believe that our results, based on internal comparisons within the cohort, are unlikely to differ between those who attended and those who did not. We have no information about what aspect of maternal malnutrition stimulated compensatory placental growth. In Finland, as in other northern European countries, the long winters brought shortages of fruit and vegetables. In addition, there were widespread food shortages around the time of the Second World War, when our cohort was born [28].

## 5. Conclusions

We have found that a large placental surface area is associated with a high body fat percentage and an increased risk of being overweight in adult life. We suggest that the enlarged surface is the result of expansion of the placental surface to compensate for fetal malnutrition in midgestation. The association between the placental surface area and adiposity was only found in people with the Pro12Pro genotype of the *PPAR*γ*2 *gene. We suggest that there is an interplay between nutritional factors and genes at the placental level, which is affecting the later risk for obesity.

## Figures and Tables

**Table 1:** Measurements of size at birth and in adult life, and frequency of PPAR*γ*2 genotype according to gender.

	Males (*n* = 927)		Females (*n* = 1075)	
	Mean	St deviation	Mean	St deviation
Measurements at birth				
Birth weight (g)	3476	500	3353	465
Placental weight (g)	655	124	643	120
Maximal placental diameter (cm)	19.5	2.3	19.3	2.2
Lesser placental diameter (cm)	17.0	2.2	16.8	2.2
Placental surface area (cm^2^)	262	58	257	58

Measurements in adult life				
Age (years)	61.5	2.8	61.5	3.0
Height (cm)	176.8	6.0	163.2	5.7
Weight (kg)	86.2	14.3	73.8	13.8
Body mass index (BMI, kg/m^2^)	27.5	4.2	27.7	5.0
Body fat percentage (%)	23.8	6.0	33.9	6.9
Lean body mass (kg)	65.0	7.9	47.8	5.7
Overweight (BMI ≥ 25 kg/m^2^, %)	74.0		67.7	

PPAR*γ*2 genotype				
Pro/Pro (%)	68.5		67.9	

**Table 2:** Odds ratios (95% confidence interval) for overweight and regression coefficients for percent body fat and lean body mass in relation to birth weight and placental size.

	Overweight*	Body fat (%)^+^	Lean body mass (kg)^+^
Birth weight (z)	1.13 (1.03 to 1.25)	0.03 (−0.26 to 0.32)	1.66 (1.36 to 1.95)
*P *for trend	0.01	0.8	<0.001
Ponderal index (z)	1.12 (1.01 to 1.23)	0.28 (−0.01 to 0.57)	0.40 (0.10 to 0.71)
*P *for trend	0.03	0.05	0.008
Placental weight (z)	1.07 (0.97 to 1.18)	0.02 (−0.28 to 0.31)	0.99 (0.69 to 1.29)
*P *for trend	0.2	0.9	<0.001
Max placental diam (z)	1.04 (0.94 to 1.14)	0.00 (−0.30 to 0.29)	0.64 (0.34 to 0.95)
*p *for trend	0.5	1.0	<0.001
Lesser placental diam (z)	1.07 (0.97 to 1.18)	0.09 (−0.20 to 0.39)	0.69 (0.39 to 1.00)
*P *for trend	0.2	0.5	<0.001
Placental area (z)	1.05 (0.95 to 1.15)	0.05 (−0.24 to 0.34)	0.69 (0.39 to 0.99)
*P *for trend	0.4	0.7	<0.001

*Odds ratios from multiple logistic regressions, including age and gender, with overweight as outcome.

^+^Coefficients from multiple linear regressions, including age and gender, with body fat percent or lean body mass as outcome.

**Table 3:** Odds ratios (95% confidence interval) for overweight and regression coefficients for percent body fat and lean body mass in relation to birthweight and placental size, according to mother's height. People whose mothers were >160 cm are included in the table.

	Overweight*	Body fat (%)^+^	Lean body mass (kg)^+^
Birth weight (z)	1.18 (1.02 to 1.35)	0.02 (−0.39 to 0.43)	1.54 (1.15 to 1.93)
*P *for trend	0.02	0.9	<0.001
Ponderal index (z)	1.15 (1.00 to 1.32)	0.28 (−0.14 to 0.69)	0.41 (0.01 to 0.82)
*P *for trend	0.05	0.2	0.04
Placental weight (z)	1.06 (0.93 to 1.22)	0.15 (−0.26 to 0.56)	0.90 (0.51 to 1.29)
*P *for trend	0.4	0.5	<0.001
Max placental diam (z)	1.02 (0.89 to 1.16)	−0.02 (−0.43 to 0.38)	0.45 (0.06 to 0.84)
*P *for trend	0.8	0.9	0.02
Lesser placental diam (z)	0.99 (0.86 to 1.13)	−0.21 (−0.61 to 0.20)	0.48 (0.09 to 0.88)
*P *for trend	0.9	0.3	0.02
Placental area (z)	0.98 (0.86 to 1.13)	−0.14 (−0.55 to 0.27)	0.47 (0.08 to 0.87)
*P *for trend	0.8	0.5	0.02
Tall mothers (HEIGHT > 160 cm)			
Birth weight (z)	1.11 (0.94 to 1.31)	0.21 (−0.29 to 0.71)	1.46 (0.95 to 1.97)
*P *for trend	0.2	0.4	<0.001
Ponderal index (z)	1.12 (0.96 to 1.32)	0.44 (−0.01 to 0.90)	0.47 (0.00 to 0.95)
*P *for trend	0.2	0.06	0.05
Placental weight (z)	1.06 (0.90 to 1.24)	0.01 (−0.47 to 0.49)	0.83 (0.33 to 1.32)
*P *for trend	0.5	1.0	0.001
Max placental diam (z)	1.06 (0.89 to 1.25)	0.12 (−0.39 to 0.62)	0.75 (0.23 to 1.28)
*P *for trend	0.5	0.6	0.005
Lesser placental diam (z)	1.18 (1.00 to 1.38)	0.58 (0.10 to 1.07)	0.65 (0.14 to 1.15)
*P *for trend	0.05	0.02	0.01
Placental area (z)	1.12 (0.95 to 1.32)	0.40 (−0.10 to 0.89)	0.71 (0.21 to 1.23)
*P *for trend	0.2	0.1	0.006

*Odds ratios from multiple logistic regressions, including age and gender, with overweight as outcome.

^+^Coefficients from multiple linear regressions, including age and gender, with body fat percent or lean body mass as outcome.

**Table 4:** Odds ratios (95% confidence interval) for overweight and regression coefficients for percent body fat and lean body mass in relation to birthweight and placental size, in the offspring of tall mothers (height >160 cm) who were carriers of the Pro12Pro genotype.

	Overweight*	Body fat (%)^+^	Lean body mass (kg)^+^
Birth weight (z)	1.20 (0.98 to 1.47)	0.41 (−0.19 to 1.01)	1.48 (0.87 to 2.09)
*P* for trend	0.08	0.2	<0.001
Ponderal index (z)	1.18 (0.97 to 1.43)	0.32 (−0.28 to 0.91)	0.61 (0.00 to 1.21)
*P* for trend	0.1	0.3	0.05
Placental weight (z)	1.18 (0.97 to 1.44)	0.28 (−0.31 to 0.87)	0.99 (0.39 to 1.60)
*P* for trend	0.1	0.4	0.001
Max placental diam (z)	1.28 (1.03 to 1.59)	0.51 (−0.14 to 1.16)	0.97 (0.30 to 1.64)
*P* for trend	0.02	0.1	0.005
Lesser placental diam (z)	1.34 (1.10 to 1.64)	0.85 (0.24 to 1.46)	1.03 (0.40 to 1.66)
*P* for trend	0.004	0.006	0.001
Placental area (z)	1.37 (1.10 to 1.71)	0.77 (0.12 to 1.42)	1.09 (0.42 to 1.75)
*P* for trend	0.005	0.02	0.001
Pro12Ala & Ala12Ala			
Birth weight (z)	0.93 (0.68 to 1.28)	−0.22 (−1.11 to 0.66)	1.41 (0.48 to 2.35)
*P* for trend	0.7	0.6	0.003
Ponderal index (z)	1.06 (0.81 to 1.40)	0.89 (0.18 to 1.61)	0.31 (−0.48 to 1.09)
*P* for trend	0.7	0.01	0.4
Placental weight (z)	0.85 (0.64 to 1.12)	−0.56 (−1.37 to 0.24)	0.50 (−0.37 to 1.37)
*P* for trend	0.3	0.2	0.3
Max placental diam (z)	0.75 (0.56 to 0.99)	−0.58 (−1.37 to 0.20)	0.41 (−0.44 to 1.26)
*p* for trend	0.04	0.1	0.3
Lesser placental diam (z)	0.88 (0.66 to 1.16)	−0.05 (−0.84 to 0.73)	−0.15 (−1.00 to 0.69)
*P* for trend	0.4	0.9	0.7
Placental area (z)	0.80 (0.61 to 1.04)	−0.27 (−1.02 to 0.49)	0.12 (−0.69 to 0.93)
*P *for trend	0.1	0.5	0.8

*Odds ratios from multiple logistic regressions, including age and gender, with overweight as outcome.

^+^Coefficients from multiple linear regressions, including age and gender, with body fat percent or lean body mass as outcome.
